# The counterintuitive effect of multiple injuries in severity scoring: a simple variable improves the predictive ability of NISS

**DOI:** 10.1186/1757-7241-19-26

**Published:** 2011-04-19

**Authors:** Stefano Di Bartolomeo, Chiara Ventura, Massimiliano Marino, Francesca Valent, Susanna Trombetti, Rossana De Palma

**Affiliations:** 1 Anaesthesia and ICU S.M.M. Hospital, Udine/Regional Health Agency of Emilia-Romagna, Bologna, Italy; 2 Regional Health Agency of Emilia-Romagna, Bologna, Italy; 3 Institute of Hygiene and Epidemiology, University Hospital, Udine, Italy

**Keywords:** (MESH): Wounds and Injuries, Trauma Severity Index, Registries, Multiple Trauma

## Abstract

**Background:**

Injury scoring is important to formulate prognoses for trauma patients. Although scores based on empirical estimation allow for better prediction, those based on expert consensus, e.g. the New Injury Severity Score (NISS) are widely used. We describe how the addition of a variable quantifying the number of injuries improves the ability of NISS to predict mortality.

**Methods:**

We analyzed 2488 injury cases included into the trauma registry of the Italian region Emilia-Romagna in 2006-2008 and assessed the ability of NISS alone, NISS plus number of injuries, and the maximum Abbreviated Injury Scale (AIS) to predict in-hospital mortality. Hierarchical logistic regression was used. We measured discrimination through the C statistics, and calibration through Hosmer-Lemeshow statistics, Akaike's information criterion (AIC) and calibration curves.

**Results:**

The best discrimination and calibration resulted from the model with NISS plus number of injuries, followed by NISS alone and then by the maximum AIS (C statistics 0.775, 0.755, and 0.729, respectively; AIC 1602, 1635, and 1712, respectively). The predictive ability of all the models improved after inclusion of age, gender, mechanism of injury, and the motor component of Glasgow Coma Scale (C statistics 0.889, 0.898, and 0.901; AIC 1234, 1174, and 1167). The model with NISS plus number of injuries still showed the best performances, this time with borderline statistical significance.

**Conclusions:**

In NISS, the same weight is assigned to the three worst injuries, although the contribution of the second and third to the probability of death is smaller than that of the worst one. An improvement of the predictive ability of NISS can be obtained adjusting for the number of injuries.

## Background

The importance of injury scoring is universally recognized and the literature on the subject is immense. Although the scores based on empirical estimation - e.g. Trauma Mortality Prediction Model [1] - are finally gaining popularity because they show better prediction [1-3], those based on the expert consensus of the Abbreviated Injury Scale (AIS) [4] lexicon are still widely used. One of the most popular is the New Injury Severity Score (NISS), which is generally recommended as an improvement over the venerable Injury Severity Score (ISS) [5-10].

The role of multiple injuries in outcome prediction and scoring is important because the majority of patients have more than one injury. For example, only 38.3% of patients of the American National Trauma Data Bank (NTDB) sustained a single injury [11]. The way different scores account for the combined effects of multiple injuries varies widely and is a controversial subject [12,13] that has begun to be elucidated only recently [14]. The last findings seem to suggest that the impact of several injuries on mortality is actually lower than the sum of impacts of the individual injuries. On the contrary, NISS is the simple sum of the three most serious AIS scores squared.

In this study we describe how, in accordance with the current knowledge, the addition of a simple variable that quantifies the number of injuries significantly improves the predictive ability of NISS for mortality.

## Methods

This study was conducted using data from the trauma registry of the region Emilia-Romagna (RRTG). Emilia-Romagna is an Italian region with about 4 million inhabitants where a trauma system was instituted in 2006. This system is based on three hubs with a defined area of competence that receive patients from scene and other hospitals according to agreed protocols via a pre-hospital Emergency Medical Service. The RRTG started in October 2006 and receives data from the three hubs and seven additional hospitals. The inclusion criteria are traumatic injuries with ISS>15 or admission to intensive care (ICU). ICU admission is decided by clinical judgment and there are no standard criteria. Patients dead on arrival or early in the Emergency Room are recorded in a separate database - not considered by the present study - because they often lack important information like injury severity. The RRTG collects information on demographics, injury, pre-hospital and hospital clinical course and outcome. Injury severity is coded according to the AIS version '98 by one trained coder per hospital. The training was self-managed by regional authorities, with no official certification by Association for the Advancement of Automotive Medicine.

All 3754 cases of the years 2007-2009 were considered for inclusion. All of the cases of 2 hospitals (n = 1180) had to be excluded, because these hospitals recorded only the ISS and not the AIS codes of each lesion. Patients with burns, asphyxia or drowning and those with age <1 year (n = 86) were also excluded. These exclusions are habitual in studies on trauma mortality prediction modeling because severity indexes may perform differently with these injuries [15] and because the cut-offs of physiologic variables chosen for adults may not apply to infants. The final number of cases was 2488.

A variable (num_inj) expressing the number of AIS-coded injuries sustained by the patients was created, with three categories: one, two, and three or more injuries. The predictive abilities of NISS alone and NISS + num_inj were assessed and compared. The largest severity measure taken from a patient's set of AIS codes (max AIS) was also computed and its discrimination ability quantified and compared. Hierarchical logistic regression models were built, with in-hospital mortality as the outcome variable and single hospitals as the random effect (random intercept). The interaction between NISS and num_inj was also tested with both Wald statistics and the Likelihood Ratio test. The predictive ability was assessed according to discrimination and calibration. Discrimination was measured with the C statistics, also known as area under the ROC curve. The significance of the differences among ROC areas was also assessed [16]. Calibration was assessed with the Hosmer-Lemeshow (HL) goodness of fit test and Akaike's information criterion (AIC), the lower the better. Both the HL C statistics (based on equally sized groups) and HL H statistics (based on fixed cut-points of the predictions) were calculated. The groups were 10 for the C statistics and between eight and ten for the H statistics, depending on the range of predictions (the cut-points for the predicted probability of death were 10%, 20%... etc.). Because it is recognized that a standard and agreed measure of calibration does not exist [17], we present calibration also by calibration curves. For simplicity, only curves of equally sized groups for simple and complete models with NISS and NISS+num_inj are shown.

A second set of models also including age (continuous), gender (categorical), and mechanism of injury (categorical) were assessed and compared. Finally, a set of models further completed with physiological information - motor component of Glasgow Coma Scale (categorical) and systolic blood pressure (categorical) - were evaluated. The detailed description of these variables is shown in table 1. The categorization of some variables is somewhat different from the Utstein recommendations [18] because we grouped some categories that had no or few cases. In addition, one extra category was introduced (systolic blood pressure >179 mmHg) because a significant effect on mortality was noticed during model development. In all models the best transformation for continuous variables age and NISS was determined with fractional polynomial transformation. Max AIS and num_inj were treated as nominal, i.e. using dummy or indicator variables.

**Table 1:** Characteristics of the population.

Characteristics	Survivors (n = 2174)	Non survivors (n = 314)	Total (2488)	Difference between survivors and non-survivors*
Age (n = 2488), mean ± SD, median (range)	44.0 ± 21.4, 41 (1-93)	61.5 ± 23.9, 70 (1-98)	46.3 ± 22.5, 43 (1-98)	p < 0.01
Gender (n = 2488), No of males (%)	1634 (75.2)	221 (70.4)	1855 (74.6)	p = 0.06
Mechanism of injury, No (%)				p < 0.01
Traffic	1509 (69.4)	180 (57.3)	1689 (67.9)	
Fall	465 (21.4)	104 (33.1)	569 (22.9)	
Penetrating	28 (1.3)	9 (2.9)	37 (1.5)	
Other	144 (6.6)	19 (6.1)	163 (6.5)	
Missing or unknown	35 (1.1)	2 (0.6)	30 (1.2)	
NISS (n = 2488), mean ± SD, median (range)	30.00 ± 13.6, 27 (1-75)	44.33 ± 18.4, 43 (1-75)	31.81 ± 15.0, 29 (1-75)	p < 0.01
Motor component of GCS, No (%)				p < 0.01
6 - Obeys	1411 (64.9)	101 (29.6)	1512 (60.8)	
5 - Localizes	329 (15.1)	37 (11.8)	366 (14.7)	
4 - Withdraws	128 (5.9)	30 (9.5)	158 (6.3)	
3 - Decorticate flexion	77 (3.5)	21 (6.7)	98 (3.9)	
2 - Extensor response	65 (2.9)	25 (8.0)	90 (3.6)	
1 - Nil	121 (5.6)	93 (29.6)	214 (8.6)	
missing	43 (2.0)	7 (2.2)	50 (2.0)	
Systolic Blood Pressure, No (%)				p <0.01
180-max	88 (4.1)	42 (13.4)	130 (5.2)	
90-179	1810 (83.3)	179 (57.0)	1989 (79.9)	
50-89	191 (8.8)	72 (22.9)	263 (10.6)	
1-49	8 (0.4)	8 (2.5)	16 (0.6)	
missing	77 (3.5)	13 (4.1)	90 (3.6)	
ICU admission, No (%)	1911 (87.9)	310 (98.7)	2221 (89.3)	p < 0.01
ICU stay (days), mean (median)	8.91 (5)	5.78 (2)	8.47 (4)	p < 0.01
Hospital stay (days), mean (median)	27.29 (16)	8.61 (2)	24.68 (14)	p < 0.01
ISS>15, No (%)	1798 (82.7)	289 (92.0)	2086 (83.9)	p < 0.01
ICU stay (days), mean, median	10.04, 6	5.81, 2	9.38, 5	p < 0.01
Hospital stay (days), mean, median	26.43, 15	7.55, 2	23.82, 13	p < 0.01
Number of injuries, No (%)				p = 0.75
1	210 (9.7)	30 (9.5)	240 (9.6)	
2	267 (12.3)	34 (10.8)	301 (12.1)	
3 or more	1696 (78.0)	250 (79.6)	1946 (78.2)	
Mortality, No (%)	/	/	314 (12.6)	/

* Kruskal-Wallis test for continuous variables and chi-square test for categorical variables

A binary variable expressing whether the two worst injuries belong to the same AIS region or not was also tested in combination with NISS and num_inj.

Missing data were treated with casewise exclusion (0, 34, and 115 exclusions respectively in simple, augmented, and complete models). All the analyses were conducted using STATA 10.

Because of the observational design of the study and the anonymity of the final database, neither patient consent nor approval of ethical committee was necessary.

## Results

Table 1 shows the characteristics of the 2488 patients by survival status.

The models' performances are shown in table 2. As for discrimination, the best to worst hierarchy was invariably 1) NISS with num_inj 2) NISS alone 3) max AIS. The difference between the former two models was significant (p < 0.05) for simple models and for models augmented with demographics and mechanism of injury. In models completed with physiological variables the size of this difference decreased and was only borderline significant (p = 0.09). However, in severely injured patients (NISS>15) the difference approached significance even in complete models (p = 0.05). Calibration measured by HL statistics was better for models with NISS+num_inj. In general it was also better for NISS alone than max AIS, though in a few instances this order was reversed.

**Table 2:** Models' Performances

Model	C statistics (95% CI)	P of C statistics comparison*	Hosmer-Lemeshow C statistics	Hosmer-Lemeshow H statistics	Akaike's information criterion
*Simple*					
MaxAIS	0.729 (0.699-0.758)	/	11.52 p = 0.24	228.68 p < 0.01	1712
NISS	0.755 (0.726-0.784)	0.02	14.69 p = 0.14	7.12 p = 0.52	1635
NISS + num_inj	0.775 (0.745-0.804)	0.03	9.03 p = 0.52	10.32 p = 0.24	1602
*Augmented*†					
MaxAIS	0.841 (0.820-0.862)	/	11.96 p = 0.28	18.66 p = 0.04	1542
NISS	0.865 (0.844-0.886)	<0.01	7.47 p = 0.68	17.51 p = 0.06	1352
NISS + num_inj	0.874 (0.855-0.894)	0.01	7.21 p = 0.72	10.27 p = 0.41	1331
*Complete*‡					
MaxAIS	0.890 (0.872-0.909)	/	10.69 p = 0.38	12.71 p = 0.24	1234
NISS	0.898 (0.880-0.916)	0.06	5.50 p = 0.85	15.87 p = 0.10	1174
NISS + num_inj	0.901 (0.884-0.919)	0.09	4.00 p = 0.94	9.05 p = 0.52	1167
*Complete*‡, *NISS>15*					
MaxAIS	0.888 (0.868-0.907)	/	7.22 p = 0.70	20.79 p = 0.02	1165
NISS	0.897 (0.879-0.916)	0.03	6.92 p = 0.73	19.14 p = 0.03	1105
NISS + num_inj	0.901 (0.883-0.919)	0.05	5.76 p = 0.83	13.68 p = 0.18	1098

* comparison with the preceding model in the table

num_inj = an indicator variable expressing the number of injuries (1,2,3+)

†augmented with age, gender and mechanism of injury

‡completed with the above variables plus systolic blood pressure and motor component of Glasgow Coma Scale

**Figure 1:**
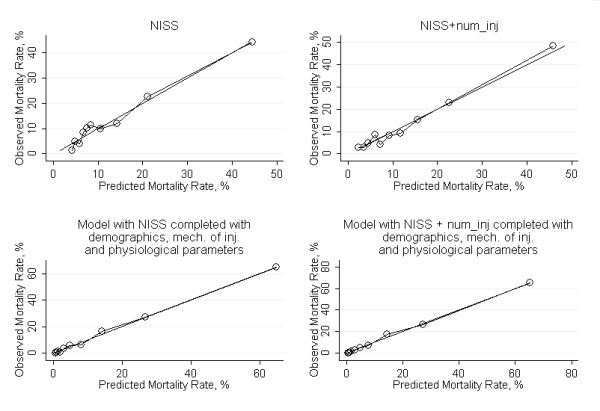
**Calibration curves**.

Figure 1 shows the calibration curves. The straight line represents the identity between observed and predicted mortality; it can be seen that there are no conspicuous differences between models with NISS and those with NISS + num_inj.

Table 3 shows the logistic regression parameters of num_inj. The risk of death adjusted for NISS was significantly lower both in patients with two injuries and in those with three, as compared to those with one. The interaction between num_inj and NISS was not significant (p of both Wald and LR test >0.1).

**Table 3:** Regression coefficients of the variable expressing the number of injuries

Predictor	Odds Ratio	Std. Error	z	P of Wald test	95% CI
2 injuries vs. 1 injury	0.520	0.146	-2.33	0.02	0.300-0.902
3 injuries vs. 1 injury	0.174	0.044	-6.92	<0.01	0.106-0.286

The model includes NISS with fractional polynomial transformation and the dependent variable is mortality

The odds ratio of the variable measuring whether the two worst injuries belong to the same AIS region was 1.19 (95% CI 0.899-1.582, p = 0.22) in the model with NISS and num_inj. Its inclusion virtually did not change the parameters of the other two variables. The interaction with num_inj was not significant either.

Table 4 displays the mortality of the three most numerous groups of patients with similar NISS scores but different number of injuries.

**Table 4:** Mortality of patients with similar NISS and different number of injuries

NISS	Mortality (%)		
	**1 injury**	**2 injuries**	**3 or more injuries**
8-9	6/70 (8.57)	1/15 (6.67)	0/12 (0)
14-17	4/66 (6.06)	0/12 (0)	5/164 (3.05)
24-26	19/75 (25.33)	3/47 (6.38)	2/62 (3.23)

## Discussion

We found that the predictive ability of models using NISS as injury-severity index is increased by the addition of a variable that summarizes the number of AIS-coded injuries sustained by the patients. The relationship between multiple injuries is fundamental in severity scoring because, as already said, the majority of patients are multiply injured. With hindsight, all the expert consensus and AIS-based scores devised so far oscillated between different ways to account for the following three factors: the contribution of multiple injuries, the weight assigned to injuries other than the worst, and the contribution of the anatomic regions. ISS postulated that the three most severe injuries determine the risk. At the same time it assigned a considerable discounting to additional injuries by disregarding altogether those of the same region. This rule factored in the importance of belonging to the same or different anatomic regions. NISS maintained the principle of considering the three worst injuries, but abolished any form of discounting and consideration for the regions. Essentially, it maximized the role of multiple injuries by giving to the second and third injury the same importance of the first one. The studies showing that max AIS predicts better than NISS and ISS [11,19,20] brought the focus again on the minimal importance of injuries other than the worst one: these lesions are disregarded altogether. The Anatomic Profile (AP) [21], instead, accounts for all three factors in a sophisticated way; the AP includes all the serious injuries in a given body region and weights head and torso injuries more heavily than those in other body regions. It is difficult to condense decades of literature and say which score and underlying method of computing multiple lesions proved best. In general, AP gave most often the best performances, though did not gain popularity for its complexity, while the ranking of the other simpler scores reversed among different studies (e.g. max AIS resulted worse than ISS and NISS in some cases [13,22], NISS was not always confirmed better than ISS [19]). The case-mix upon which any method is tested unarguably plays a role, e.g. as injury severity burden worsens, the worst-injury-only scores are penalized [11].

Another line of research has revealed that patients with identical ISS/NISS scores resulting from different underlying AIS triplets carry quite different mortality risks [23-25]. The researchers focused on the fact that triplets containing the highest AIS score (e.g. 3-0-0 vs. 2-2-1) invariably carried the highest mortality [25]. In the light of our study, it can also depend on the fact that these triplets were also those with the smallest number of injuries for a same ISS/NISS score.

The recent research on new empirical severity scores [1,13] has shed further light on the role of multiple injuries by carefully quantifying their effect on outcome. In these papers Osler and colleagues showed that it is worth considering up to five injuries, provided that those additional to the worst are carefully weighted. The weight they found for the additional injuries is approximately half that of the worst one. The authors' clinical interpretation of this statistical finding is biologically plausible: although further injuries increase the probability of death, their contribution to the likelihood of death is reduced. They also found that further discounting of predicted mortality results if the 2 worst injuries occur in the same body region (odds ratio for the two worst injuries belonging to the same AIS region: 0.87, p < 0.01).

The behavior of the variable num_inj in our study seems to confirm most of the previous findings. When adjusting for NISS, having two and more than two injuries instead of one lowers the risk of death to approximately one half and one fifth, respectively (table 3). Table 4 is a practical exemplification of the statistical properties of num_inj reported in table 3.

NISS tends to overestimate the risk of death in case of multiple injuries because it does not apply any discounting to the second and third worst lesions. On the other hand, considering only the most severe injury in absolute (max AIS) or by region (ISS) may cause an underestimation of the same risk, as shown by the lowest discrimination of max AIS in our study and by the studies reporting worse performances of max AIS or ISS compared to NISS [5-10,19,26]. The adjustment for the number of injuries may address this limitation of NISS in a maybe unorthodox but seemingly efficacious way, preserving the score's inherent simplicity and consideration for multiple injuries. The downside is that a regression model is needed to incorporate this variable, while clinicians use injury scores also as handy, immediate information. However, multivariate models are often used for research and quality assessment and a variable that is both easy to collect and capable of improving the models could be of interest.

An inevitable limitation of num_inj is that it does not discriminate between degrees of injuries. If the second injury is trivial - e.g. a patient with two lesions with AIS severity of 5 and 1 - the increase in prediction given by num_inj is likely to be negligible. A possible refinement, especially for severely injured populations, could be the adoption of a severity threshold (e.g. ≥2) for AIS codes to be included in num_inj.

The fact that in models completed with physiological variables the gain in prediction brought by num_inj is not statistically significant should not be seen as a serious limitation. Physiological variables describe the actual clinical status of the patient that is an important predictor of final outcome. So it is reasonable that they may 'refine' more imperfect anatomic predictors. Yet physiological information is not always available. Moreover, it is possible that in a larger database the models with num_inj could perform significantly better also if completed with physiological variables and we encourage further research.

In our case-mix max AIS performed worse than both NISS and NISS + num_inj. This leaves one question unanswered, i.e. how NISS with num_inj would perform in a population (presumably less-severely injured than ours) where max AIS works better than NISS. Since we tested the interaction of num_inj with NISS and found it non-significant, it would seem that the effect of num_inj be the same across the whole spectrum of injury severity, but, again, more research is needed to answer the question.

Contrary to the findings of Osler et al. we could not show any effect of the two worst injuries belonging to the same AIS region or not. This contradicts a major tenet in the definitions of polytrauma, i.e. that the involvement of different regions increases mortality [27]. Unfortunately we have no explanation for this counterintuitive finding. The term AIS region refers to the nine anatomic regions of the AIS, but is sometimes confused in literature with ISS region, which is their re-arrangement into six, less homogeneous, regions used for ISS computation. We also tested this latter variable, with results similar to those mentioned (data not reported).

This study has some limitations. The number of cases is small and further studies are awaited to confirm or dispute our findings. Moreover, the cases with ISS<16 are not representative of their entire population because RRTG enrolls them only if admitted to ICU. We chose to include in this study all eligible RRTG patients, subtracting to homogeneity, in order to maximize the number of cases. We recognize, however, that this limits the generalizability of our conclusions, calling for further studies in larger populations.

A further limitation is that although we used NISS for selecting patients according to severity (table 2), the RRTG used ISS to define the inclusion threshold for patients not admitted to ICU. Because ISS underestimates severity compared to NISS, the exclusion of some cases with NISS>15 is likely to have occurred. This has probably made this group not representative of the real population with NISS>15, but is unlikely to have influenced our findings.

Another possible criticism is that our outcome was hospital mortality instead of 30-day mortality, recommended by the Utstein template [18]. Unfortunately we were obliged to do so because data on 30-day mortality become available for research with a great delay in our setting. They were available only for the years 2007 and 2008, and suggested that 30-day mortality is probably lower in our setting (9.9 vs. 12.4). However, hospital mortality has been commonly used in most of the cited studies.

Because the predictive performances of models were determined on the same sample of subjects that was used to construct the model, they were probably overestimated. However, such an overestimation would be common to all models and therefore should not affect the relative comparisons between them, the main goal of the study.

## Conclusions

In NISS, the same weight is assigned to the three worst injuries, although the contribution of the second and third to the likelihood of death is smaller than that of the worst one. An improvement of the predictive ability of NISS can be obtained adjusting for the number of injuries.

## Competing interests

The authors declare that they have no competing interests.

## Authors' contributions

SDB conceived the study, carried out the statistical analyses and drafted the manuscript. CV and MM participated in the statistical analyses. FV participated in the conception of the study, participated in the statistical analysis and revised the manuscript. ST helped to draft the manuscript. RDP revised it critically for important intellectual content. All authors read and approved the final manuscript.
